# Differences in net global warming potential and greenhouse gas intensity between major rice-based cropping systems in China

**DOI:** 10.1038/srep17774

**Published:** 2015-12-02

**Authors:** Zhengqin Xiong, Yinglie Liu, Zhen Wu, Xiaolin Zhang, Pingli Liu, Taiqing Huang

**Affiliations:** 1 Jiangsu Key Laboratory of Low Carbon Agriculture and GHG Mitigation, College of Resources and Environmental Sciences, Nanjing Agricultural University, Nanjing 210095, China

## Abstract

Double rice (DR) and upland crop-single rice (UR) systems are the major rice-based cropping systems in China, yet differences in net global warming potential (NGWP) and greenhouse gas intensity (GHGI) between the two systems are poorly documented. Accordingly, a 3-year field experiment was conducted to simultaneously measure methane (CH_4_) and nitrous oxide (N_2_O) emissions and changes in soil organic carbon (SOC) in oil rape-rice-rice and wheat-rice (representing DR and UR, respectively) systems with straw incorporation (0, 3 and 6 t/ha) during the rice-growing seasons. Compared with the UR system, the annual CH_4_, N_2_O, grain yield and NGWP were significantly increased in the DR system, though little effect on SOC sequestration or GHGI was observed without straw incorporation. Straw incorporation increased CH_4_ emission and SOC sequestration but had no significant effect on N_2_O emission in both systems. Averaged over the three study years, straw incorporation had no significant effect on NGWP and GHGI in the UR system, whereas these parameters were greatly increased in the DR system, i.e., by 108% (3 t/ha) and 180% (6 t/ha) for NGWP and 103% (3 t/ha) and 168% (6 t/ha) for GHGI.

Global warming undoubtedly results from greenhouse gas (GHG) emissions^1^. Nitrous oxide (N_2_O), methane (CH_4_), and carbon dioxide (CO_2_) are three GHGs of major concern that are emitted from agricultural soils^2^; however, large amounts of carbon can also be fixed in soil-crop systems through photosynthesis^3^. The net exchange of CH_4_, N_2_O and CO_2_ in terms of CO_2_ equivalents between soils and the atmosphere comprises the net global warming potential (NGWP) of a cropping system^4^, which can also be expressed on the basis of per unit of yield (greenhouse gas intensity, GHGI)^3^.

Agriculture is an important source of CH_4_ and N_2_O^2^, accounting for 50% and 60% of total global anthropogenic CH_4_ and N_2_O emissions, respectively, in 2005^5^. Rice paddy fields have been identified as a major source of CH_4_ emission to the atmosphere; N_2_O is mainly generated by upland fields and is also produced from rice fields because of midseason drainage and moist irrigation^6^,^7^. The rice harvest in China, which averaged 30 M ha from 2010 to 2013, accounts for 18.7% of the world’s total^8^, and the total CH_4_ emissions from Chinese rice paddies are estimated to be 7.41 Tg CH_4_ year^−1^, 29.9% of the world’s total (25.5 Tg CH_4_ year^−1^)^9^. Additionally, direct N_2_O emission during the rice-growing season, which was measured at a rate of 32.3 Gg N_2_O-N in the 1990s, is responsible for 8–11% of the total N_2_O emission from Chinese croplands^10^.

Double rice (DR) and upland crop-single rice (UR) annual rotations are two major rice systems in China, with 75% of rice fields implementing these approaches^11^. Many studies have focused on CH_4_ and N_2_O emissions from UR^7^,^12^ and DR systems^13^,^14^,^15^ individually. Previous data based on pot experiments indicate that different cropping systems result in different amounts of N_2_O emission from paddy fields^16^, and individual field studies have also reported variable CH_4_ emissions among cropping systems^12^,^13^. However, no field study to date has simultaneously addressed CH_4_ and N_2_O emissions from different rice cropping systems. Moreover, to our knowledge, the differences in NGWP and GHGI between different rice cropping systems have not been documented.

Straw return has been widely recommended for agricultural ecosystems in China. Chinese agriculture produces approximately 620 Tg of crop straw every year, with an increasing trend of an annual rate of 1.4%^17^, and approximately 25% of the straw is currently returned to the field^18^. Indeed, straw incorporation is a common practice in rice production, as it helps to maintain soil quality and recycle mineral nutrients^19^. Straw incorporation also has a considerable influence on CH_4_ and N_2_O via changes in soil properties, such as the porosity, temperature and moisture^20^,^21^. In general, straw incorporation can enhance carbon sequestration, resulting in improved soil productivity and air quality, and thus offset GHG emissions from rice fields. However, a significant stimulation of CH_4_ emission due to straw incorporation in rice fields has been well documented^22^,^23^,^24^. In contrast, straw incorporation inhibits^7^,^12^ or has no significant effect^25^ on N_2_O emission from rice fields. Nonetheless, the mechanism by which straw addition affects carbon sequestration as well as CH_4_ and N_2_O emissions and GHGI in different rice cropping systems remains unknown.

Based on previous studies, we hypothesize that (1) different rice cropping systems may differ greatly in CH_4_ and N_2_O emissions due to drastic flooding periods and (2) straw incorporation may result in different influences on CH_4_ and N_2_O emissions from different rice cropping systems. To test these hypotheses, a field experiment was established to measure CH_4_ and N_2_O emissions and SOC changes between the two major rice cropping systems in China. The objectives were to gain insight into the differences in grain yield, NGWP and GHGI between UR and DR systems as affected by straw application.

## Results

### CH_4_ emission

**Figure 1:**
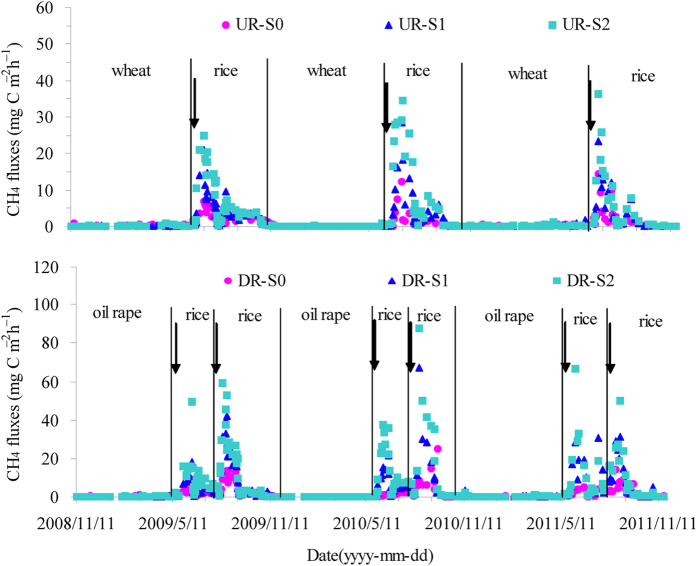
Seasonal variation of CH_4_ fluxes in the three annual cycles of UR and DR systems from November 2008 to November 2011. The arrow indicates straw incorporation.

Analysis of variance (ANOVA) indicates that annual CH_4_ emission depended strongly on the cropping system, straw incorporation, and their interactions (Table 1). Inter-annual variation was also observed (Table 1). In the UR system, similar seasonal patterns of net CH_4_ flux were observed for all treatments. The net CH_4_ flux was significant during the rice-growing season but was negligible during the wheat-growing season (Fig. 1, Table 2), ranging from −1.45 to 36.2 mg C m^−2^ h^−1^ during the three annual rotations. In addition, the net CH_4_ flux increased after rice transplantation and decreased dramatically during the midseason drainage; after reflooding, the flux increased again to a low emission peak and then decreased gradually to a negligible amount until harvest. In the DR system, all plots served as minor sinks or sources for atmospheric CH_4_ during the oil rape-growing season (Table 2), and all plots served as net sources of atmospheric CH_4_ during the early and late rice-growing seasons (Table 2). The patterns of CH_4_ flux observed during the early and late rice-growing seasons were similar to those of the rice-growing season in the UR system. Compared with the UR-S0 plot (104 kg CH_4_ ha^−1^ year^−1^), the annual CH_4_ emission increased significantly by 76.9% in the DR-S0 treatment (185 kg CH_4_ ha^−1^ year^−1^).

Straw incorporation significantly stimulated CH_4_ emission. The highest CH_4_ fluxes, i.e., 24.8, 34.5 and 36.2 mg C m^−2^ h^−1^, were observed in 2009, 2010 and 2011, respectively, in the UR-S2 plot. The annual CH_4_ emissions averaged over the three years were 104, 208 and 303 kg CH_4_ ha^−1^ year^−1^ for the UR-S0, UR-S1 and UR-S2 plots, respectively (Table 3). The average annual CH_4_ emissions were 99.7% and 191% higher in the UR-S1 and UR-S2 plots, respectively, than in the UR-S0 plot. In comparison to UR-S1, the annual CH_4_ emission was significantly increased in the UR-S2 treatment (by 45.5%). Similar to the UR system, the highest CH_4_ fluxes were observed in the DR-S2 plot, i.e., 59.0, 87.4 and 66.4 mg C m^−2^ h^−1^ in 2009, 2010 and 2011, respectively. The annual CH_4_ emissions averaged over the three years were 185, 469 and 702 kg CH_4_ ha^−1^ year^−1^ for the DR-S0, DR-S1 and DR-S2 plots, respectively. Compared with the DR-S0 plots, annual CH_4_ emissions were significantly increased by 150% and 280% in DR-S1 and DR-S2, respectively.

Furthermore, straw incorporation enhanced the differences between the DR and UR systems (Table 3). The increase due to straw incorporation in the DR system was 150% and 280% for DR-S1 and DR-S2, respectively, obviously higher than the 99.7% and 191% observed for UR-S1 and UR-S2, respectively in the UR system.

### N_2_O emission

**Figure 2:**
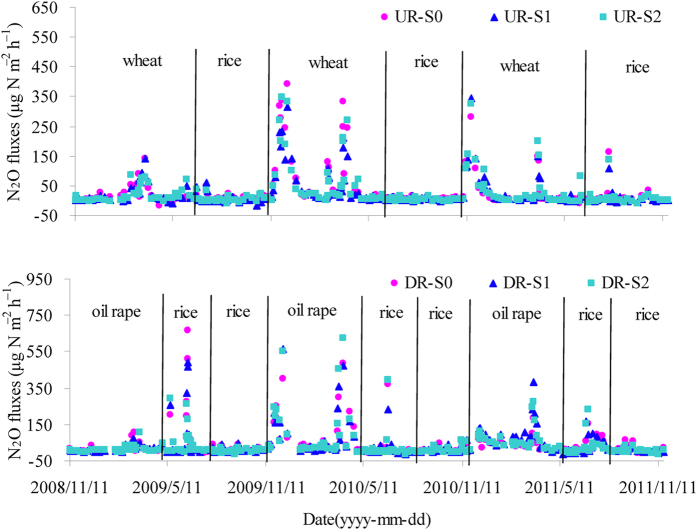
Seasonal variation of N_2_O fluxes in the three annual cycles of UR and DR systems from November 2008 to November 2011.

The majority of N_2_O emission occurred during the wheat-growing season in the UR system and the oil rape-growing season in the DR system (Fig. 2), and the N_2_O fluxes were primarily driven by fertilizer application and precipitation. No peaks of N_2_O flux were observed during the 2009 wheat- and oil rape-growing seasons because almost no precipitation occurred after the application of the basic fertilizer. However, during the rice-growing season, N_2_O flux peaks were observed in response to both N fertilizer application and midseason aeration. Straw incorporation tended to decrease N_2_O emission during the rice-growing season in both systems; however, the effects were not statistically significant (Table 2).

The annual N_2_O cumulative emissions averaged over the three years were 2.26, 2.08 and 2.19 kg N ha^−1^ year^−1^ for the UR-S0, UR-S1 and UR-S2 plots and 2.77, 3.13 and 3.04 kg N ha^−1^ year^−1^ for the DR-S0, DR-S1 and DR-S2 plots, respectively (Table 3). It is apparent that the annual N_2_O emissions were significantly increased in the DR system relative to the UR system (Table 3). Analysis of variance (ANOVA) indicated that the annual N_2_O emission was strongly dependent on the cropping system but not influenced by straw incorporation. Although inter-annual variation was observed, no interactions were found (Table 1).

### SOC sequestration

The soil organic C content was 14.6 g kg^−1^ upon establishment of the field experiment in November 2008, and SOC in the topsoil (0–20 cm) increased in all treatments over the three years of the study. After the three cycles of field experiment, the SOC contents reached 14.7 g kg^−1^, 15.7 g kg^−1^, 16.7 g kg^−1^, 15.2 g kg^−1^, 16.3 g kg^−1^ and 17.4 g kg^−1^ in UR-S0, UR-S1, UR-S2, DR-S0, DR-S1 and DR-S2, respectively. From November 2008 to November 2011, the rate of SOC increase ranged from 0.03 g C kg^−1^ yr^−1^ for the UR-S0 plot to 0.95 g C kg^−1^ yr^−1^ for the DR-S2 plot. The topsoil SOC density was estimated based on the topsoil SOC content and bulk density, with the SOC sequestration rate (SOCSR) ranging from 0.08 t C yr^−1^ for UR-S0 to 2.42 t C yr^−1^ for DR-S2 (Table 3). Compared with the UR-S0 plot, SOCSR tended to increase in the DR-S0 plot, but this effect was not statistically significant. Straw incorporation enhanced SOCSR in both the UR and DR systems, but the differences among UR-S1 and DR-S1, and UR-S2 and DR-S2 were not statistically significant (Table 3).

### Yield, NGWP and GHGI

Over the three years, the annual yields were strongly dependent on the cropping system and year as well as their interaction (Table 1). The annual yield was significantly increased in the DR-S0 plot compared with the UR-S0 plot (Table 3). However, straw incorporation had no significant effect on the seasonal grain yield, except that the late rice yield from DR-S2 was increased by 14.7% compared with DR-S0 (Table 2); straw incorporation tended to increase the annual grain yield of both systems but not to a statistically significant extent (Table 3). Relative to the UR system, the annual grain yield of the DR system was significantly increased when straw was incorporated (Table 3).

NGWP was significantly influenced by the cropping system, straw incorporation, and the year, varying significantly as a result of interactions between the cropping system and straw incorporation as well as straw incorporation and the year. GHGI also strongly depended on the cropping system, straw incorporation, and the year as well as the interaction between the cropping system and straw incorporation (Table 1). Relative to the UR system, the annual NGWP of the DR system increased markedly while no significant difference between the UR-S0 and DR-S0 treatments was observed for GHGI (Table 3). Although straw incorporation had no significant effect on the annual NGWP and GHGI of the UR system, these parameters significantly increased in the DR system, and compared with a moderate rate, the incorporation of straw at a high rate resulted in further annual NGWP and GHGI increases in the DR system (Table 3).

## Discussion

In the present study, the annual CH_4_ emission from the DR system was significantly higher than that from the UR system (Table 3). Due to the long period of flooding, double rice cropping systems emit more CH_4_ than single rice cultivation systems^26^. In fact, water management has been recognized as one of the most important practices affecting CH_4_ emission from paddy fields^27^. When plots are flooded, oxygen cannot diffuse into the soil, and strong anaerobic conditions may develop, thus favouring the growth of methanogen^28^.

In our study, annual N_2_O emissions were significantly increased in the DR system, which is not in agreement with the results of previous pot experiments^16^. However, differences between field and pot experiments may produce different results with regard to N_2_O emissions. Nonetheless, the inter-annual variation in N_2_O emissions was significant (Table 1), and the cumulative N_2_O emissions were considerably lower during the non-rice season in both the UR and DR systems in 2009 compared with 2010 and 2011 (Table 2). When soil is not maintained under flooded conditions, the water-filled pore space (WFPS) and the available N content are the two most important factors affecting N_2_O emissions^7^,^29^,^30^. In addition, high N_2_O emissions during the non-rice season generally occurred after the application of basal fertilizer and precipitation events in 2010 and 2011, consistent with a previous study^21^. Indeed, precipitation events can create suitable soil moisture conditions for N_2_O production via microbial processes^31^, yet almost no N_2_O flux peaks were observed during the non rice-growing season in both the UR and DR systems in 2009 because no precipitation occurred after the basal fertilizer application. Similar results were observed by Ma *et al.*^30^ in the same region.

Because NGWP is dominated by CH_4_ emissions in both systems, high CH_4_ emissions resulted in a significantly higher NGWP for the DR system compared with the UR system (Table 3). NGWP was also significantly higher in DR-S0 than in UR-S0, which was accompanied by an annual grain yield that was dramatically higher in DR-S0 compared with UR-S0. Consequently, there was no significant difference between the UR-S0 and DR-S0 treatments with respect to GHGI (Table 3). These two major rice cropping systems are equally appropriate for sustainable rice production on the basis of per unit of yield.

Straw incorporation significantly increases CH_4_ emissions because of the additional C that is available for methanogenesis during the rice-growing season, which has been widely demonstrated in previous studies^12^,^14^,^23^. CH_4_ emissions were highest in the S2 treatment, followed by S1 and S0, during the rice-growing seasons in both the UR and DR systems (Table 2). CH_4_ is typically produced under strictly anaerobic conditions with a low soil redox potential^27^, and the net CH_4_ flux is the balance between methanogenic and methanotrophic processes^32^. Thus, organic amendment and the water regime during the rice-growing season are the top two variables controlling CH_4_ flux^33^.

Straw incorporation tended to decrease N_2_O emissions during the rice-growing season in both the UR and DR systems (Table 2), a finding also supported by previous studies^7^,^34^. N_2_O is naturally produced in soil through nitrification and denitrification^2^, which are generally regulated by the availability of organic C and the availability and forms of N in the soil under anaerobic or aerobic conditions^35^,^36^. The observed decreases in N_2_O during the rice-growing season in the presence of straw incorporation may be explained by the following: the decomposition of crop residues with a high C:N ratio (>40) can enhance microbial N immobilization, resulting in less available N for nitrification and denitrification and consequently decreased N_2_O emissions^12^,^22^. Furthermore, our previous study proved that straw incorporation during the rice-growing season can decrease the soil redox potential (Eh) and increase the concentration of Fe^2+^, thus facilitating the further reduction of N_2_O to N_2_ and resulting in decreased N_2_O emissions^7^.

Although straw incorporation had no effect on NGWP and GHGI in the UR system, significant increases were observed in the DR system (Table 3). This finding is primarily related to the high amount of CH_4_ emissions induced by straw incorporation. In addition, straw incorporation enhanced the difference in NGWP between the UR and DR systems (Table 3) because the incorporation occurred in both the early and late rice seasons in the latter system. As the annual NGWP driven by straw incorporation outweighed the benefits of grain yield and SOCSR increases, the annual GHGI was significantly increased in DR-S1 and DR-S2 compared with UR-S1 and UR-S2, respectively. Therefore, considering the annual NGWP and GHGI, direct straw incorporation during the rice-growing season in China is beneficial for the UR system but is not a good strategy for the DR system.

## Materials and Methods

### Experimental site

A 3-year field experiment was conducted from November 2008 to November 2011 in Mo ling town, Nanjing, Jiangsu Province, China (31°52′N, 118°50′E). The site is located on the Yangtze Delta Plain, which is one of the most developed regions of China and includes portions of Jiangsu Province, Zhejiang Province and Shanghai City. Triple cropping systems with double rice seasons or double cropping systems with single rice seasons within one year are generally implemented in this region. Details of the cultivation practices for each crop season are shown in Table S1. The region is characterized by a typical subtropical climate with an annual average air temperature of 15.7°C and precipitation of 1050 mm. The daily mean air temperatures and precipitation during the experiment were collected from a nearby weather station, as shown in Fig. S1. The soil of the experimental field has a bulk density of 1.28 g cm^−3^, pH 5.7, organic C content of 14.6 g kg^−1^, and total N content of 1.32 g kg^−1^.

### Experimental treatments and field management

Two crop rotation systems were included in this experiment, i.e., a wheat-rice rotation, which represents the UR system, and an oil rape-early rice-late rice rotation, which represents the DR system. Straw (0, 3 and 6 t/ha) was incorporated during the rice season in the UR system (UR-S0, UR-S1, UR-S2) and during both the early and late rice seasons in the DR system (DR-S0, DR-S1, DR-S2) before rice transplantation (Table S2). Nitrogen fertilizer (urea) was applied at a rate of 200 kg N ha^−1^ for the early and late rice seasons and 250 kg N ha^−1^ for the other crop seasons. A total of six field experimental treatments with three replicated plots (4 m × 5 m) were established using a randomized block design. The nitrogen fertilizer (urea) was split broadcast at a ratio of 4:3:3 as a basal fertilizer and two topdressings. Phosphate and potassium fertilizers were applied uniformly as a basal fertilizer to the different treatments at 60 kg P_2_O_5_ ha^−1^ and 120 kg K_2_O ha^−1^. Field management followed the local agronomic practices, including cultivation, irrigation, fertilizer application and pest and weed control.

All field plots were drained in the winter season. Consistent with the water management of local winter crop-rice systems, flooding was initiated 2–3 days before rice transplantation and was maintained for 30–40 days until midseason drainage for one week. A final drainage event occurred approximately 15 days before rice harvesting in all treatments.

### Measurements of CH_4_ and N_2_O fluxes

CH_4_ and N_2_O emissions were measured from November 2008 to November 2011 using static opaque chambers and gas chromatography. One chamber was placed within each treatment replicate to achieve three replicate gas flux measurements for each observation time. The chamber, which was 0.5 or 1.1 m tall, having been adapted for the crop growth and plant height, covered a field area of 0.2025 m^2^ (45 × 45 cm) and was placed on a fixed PVC frame in each plot. To minimize air temperature changes inside the chamber during sampling, the chamber was wrapped with a layer of sponge and aluminium foil. For each flux measurement, four gas samples were collected from 9:00 to 11:00 am using a 25-mL syringe at 0, 10, 20, and 30 min after the chambers were placed on the fixed frames. Over the three annual cycles, CH_4_ and N_2_O fluxes were generally measured once a week in triplicate plots for all treatments, but samples were collected more frequently after a precipitation event, fertilizer application and rice transplantation.

The flux (F) of CH_4_ and N_2_O was calculated using the following equation:


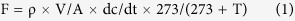


where F is the flux of greenhouse gas (mg•m^−2^•h^−1^), ρ is the density of CH_4_ (0.536 g•L^−1^) or N_2_O (1.25 g•L^−1^), V is the volume of the static opaque chamber (m^3^), A is the cover areas of the fixed PVC frame (m^2^), dc/dt is the rate at which the concentration of CH_4_ or N_2_O changes with time, and T is the temperature inside the static opaque chamber (°C).

The gas samples were analysed for CH_4_ and N_2_O concentrations using a gas chromatograph (Agilent 7890A, Shanghai, China) equipped with an electron capture detector (ECD) for N_2_O analysis and a hydrogen flame ionization detector (FID) for CH_4_ analysis (CO_2_ was first reduced by hydrogen to CH_4_ in a nickel catalytic converter at 375°C). N_2_O was separated using two stainless steel columns packed with 80–100 mesh Porapak Q. One column was 2 m long with an inner diameter of 2 mm; the other column was 3 m long with an inner diameter of 2 mm. The carrier gas was argon-methane (5%) at a flow rate of 40 ml min^−1^. The temperatures of the columns and the ECD detector were maintained at 40°C and 300°C, respectively, and the oven and FID were operated at 50°C and 300°C, respectively. The detection limits for CH_4_ and N_2_O in this study are 0.023 mg C m^−2^ h^−1^ and 1.72 μg N m^−2^ h^−1^, respectively.

### Measurements of changes in SOC

Soil samples were collected when the field experiment was initiated in November 2008 and after three years in November 2011. A composite sample for each plot was obtained by randomly collecting five or six soil cores at a depth of 20 cm (3 cm diameter) and mixing them thoroughly. Any visible roots, stones, or organic residues were removed manually after air-drying the samples at room temperature. The samples were then ground to pass through a 2-mm sieve, and a portion was subsequently ground in a porcelain mortar to pass through a 0.15-mm sieve for SOC measurement. The total SOC was analysed following wet digestion with H_2_SO_4_-K_2_Cr_2_O_7_. The minimum change in SOC that can be detected by this method is 0.01 g kg^−1^.

The soil organic carbon sequestration rate (SOCSR) was calculated as follows:


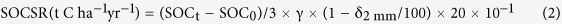


where SOC_t_ and SOC_0_ are the SOC contents measured in November 2011 and 2008, respectively, and γ and δ_2mm_ are the average bulk density (in grams per cubic centimetres) and gravel content (>2 mm) of the topsoil (0–20 cm), respectively. The sand fractions of paddy soils in China are largely negligible. The number 20 represents the thickness of the topsoil.

### Calculation of NGWP and GHGI

NGWP of a cropland ecosystem equals the total CO_2_ emission equivalents minus the change in SOC; thus, NGWP and GHGI were calculated as follows:


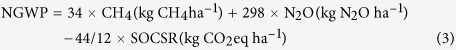



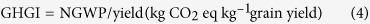


where the numbers 34 and 298 represent the IPCC factors for the conversion of CH_4_ and N_2_O to CO_2_ equivalents, respectively^1^.

### Statistical analysis

Statistical analyses were performed using JMP 9.0 (SAS Institute Inc., Cary, USA). Three-way factorial analysis of variance (ANOVA) was used to test the effect of cropping system, straw incorporation and year on annual CH_4_ and N_2_O emissions, yields, NGWP and GHGI.

## Additional Information

**How to cite this article**: Xiong, Z. *et al.* Differences in net global warming potential and greenhouse gas intensity between major rice-based cropping systems in China. *Sci. Rep.* **5**, 17774; doi: 10.1038/srep17774 (2015).

## Supplementary Material

Supplementary Materials

## Figures and Tables

**Table 1:** Three-way analysis of variance (ANOVA) results for the effects of cropping system (C), straw incorporation (S), year (Y) and their interactions on CH_4_ and N_2_O emissions, grain yield, NGWP and GHGI.

Factors	df	CH_4_(kg ha^−1^)			N_2_O (kg N ha^−1^)			Yield (t ha^−1^)			NGWP (t CO_2_ha^−1^)			GHGI		
		SS	F	P	SS	F	P	SS	F	P	SS	F	P	SS	F	P
C	1	820939	147.82	<0.001	8.83	13.05	<0.001	147.18	39.27	<0.001	643.08	92.73	<0.001	1.42	36.47	<0.001
S	2	1156794	104.14	<0.001	0.11	0.08	0.92	2.87	0.38	0.68	279.40	20.14	<0.001	0.98	12.57	<0.001
Y	2	42201	3.80	<0.05	52.66	38.91	<0.001	83.23	11.10	<0.001	103.99	7.50	<0.05	0.74	9.51	<0.001
C × S	2	229879	20.70	<0.001	0.67	0.49	0.61	0.27	0.04	0.97	229.36	16.54	<0.001	0.71	9.13	<0.001
C × Y	2	27827	2.51	0.10	0.27	0.20	0.82	100.28	13.38	<0.001	34.74	2.50	0.10	0.08	1.08	0.35
S × Y	4	63849	2.87	<0.05	2.27	0.84	0.51	10.26	0.68	0.61	76.82	2.77	<0.05	0.33	2.10	0.10
C × S × Y	4	14844	0.67	0.62	0.54	0.20	0.94	8.23	0.55	0.70	19.50	0.70	0.60	0.03	0.17	0.95
Model	17	2356334	24.96	<0.001	65.35	5.68	<0.001	352.31	5.53	<0.001	1386.88	11.76	<0.001	4.28	6.48	<0.001
Error	36	199937			22.36			134.92			249.65			1.40		

^*^Mean ± SD, different letters within a column indicate significant differences between treatments according to Tukey’s multiple range tests (*P* < 0.05).

**Table 2:** Seasonal CH_4_ and N_2_O emissions and grain yields measured in UR and DR systems from November 2008 to November 2011.

Treatment	CH_4_ (kg ha^−1^)			N_2_O (kg N ha^−1^)			Yield (t ha^−1^)		
	oil rape/wheat	early/single rice	late rice	oil rape/wheat	early/single rice	late rice	oil rape/wheat	early/single rice	late rice
2008–2009									
UR-S0	−1.06 ± 2.29	114 ± 23.8		0.99 ± 0.46	0.18 ± 0.16		5.44 ± 0.21	8.42 ± 0.24	
UR-S1	0.66 ± 2.64	220 ± 57.7		0.97 ± 0.22	0.14 ± 0.10		7.98 ± 4.73	8.14 ± 0.21	
UR-S2	−0.39 ± 0.83	305 ± 78.4		0.94 ± 0.03	0.17 ± 0.06		5.71 ± 2.17	8.42 ± 0.40	
DR-S0	3.07 ± 2.50	57.8 ± 12.5	130 ± 25.6	0.59 ± 0.46	1.03 ± 0.50	0.18 ± 0.06	2.39 ± 0.57	5.13 ± 0.40	6.52 ± 0.28
DR-S1	3.69 ± 1.40	123 ± 47.1	271 ± 48.5	0.62 ± 0.15	0.99 ± 0.09	0.14 ± 0.08	2.42 ± 0.51	5.29 ± 0.40	6.48 ± 0.31
DR-S2	3.32 ± 1.33	252 ± 40.9	364 ± 130	0.61 ± 0.13	0.90 ± 0.60	0.16 ± 0.14	2.18 ± 0.55	5.31 ± 0.34	6.99 ± 0.26
2009–2010									
UR-S0	1.74 ± 0.71	108 ± 11.3		3.63 ± 0.64	0.22 ± 0.17		2.88 ± 0.33	9.28 ± 0.78	
UR-S1	1.62 ± 1.63	231 ± 37.1		2.80 ± 0.89	0.17 ± 0.03		2.67 ± 0.59	8.19 ± 0.67	
UR-S2	4.80 ± 2.54	383 ± 49.7		3.22 ± 0.34	0.13 ± 0.06		2.42 ± 0.51	9.38 ± 1.08	
DR-S0	1.41 ± 1.47	10.5 ± 6.27	165 ± 58.0	3.75 ± 1.54	0.26 ± 0.24	0.15 ± 0.09	0.64 ± 0.24	8.66 ± 1.11	8.11 ± 0.75
DR-S1	−0.47 ± 1.20	98.8 ± 36.2	379 ± 18.8	3.84 ± 1.13	0.16 ± 0.11	0.18 ± 0.15	0.67 ± 0.17	7.70 ± 2.49	8.40 ± 0.28
DR-S2	1.45 ± 1.31	204 ± 18.6	618 ± 119	4.18 ± 2.02	0.25 ± 0.12	0.12 ± 0.12	0.51 ± 0.09	7.91 ± 0.65	9.10 ± 0.85
2010–2011									
UR-S0	6.28 ± 5.07	83.9 ± 17.5		1.39 ± 0.42	0.37 ± 0.12		4.76 ± 0.63	9.88 ± 0.92	
UR-S1	11.52 ± 9.25	161 ± 37.3		1.82 ± 0.26	0.33 ± 0.10		5.73 ± 0.59	8.44 ± 1.02	
UR-S2	6.18 ± 3.66	210 ± 41.3		1.84 ± 0.28	0.27 ± 0.19		4.60 ± 1.72	11.63 ± 3.12	
DR-S0	1.25 ± 3.80	84.1 ± 15.8	100 ± 4.16	1.53 ± 0.66	0.62 ± 0.32	0.21 ± 0.05	1.58 ± 0.40	6.89 ± 0.65	9.93 ± 2.59
DR-S1	3.34 ± 5.08	338 ± 96.4	191 ± 57.6	2.80 ± 0.66	0.51 ± 0.17	0.16 ± 0.07	1.73 ± 0.16	6.33 ± 1.18	11.53 ± 1.30
DR-S2	−1.54 ± 5.55	441 ± 67.3	224 ± 53.8	2.17 ± 0.36	0.54 ± 0.15	0.20 ± 0.13	1.63 ± 0.13	5.94 ± 1.40	12.08 ± 0.31
Average 2008–2011									
UR-S0	2.32 ± 2.53a	102 ± 10.6c		2.00 ± 0.08a	0.26 ± 0.15a		4.35 ± 0.23a	8.91 ± 0.55a	
UR-S1	4.60 ± 3.13a	204 ± 27.5b		1.86 ± 0.18a	021 ± 0.04a		5.38 ± 1.59a	8.36 ± 0.69a	
UR-S2	3.53 ± 0.49a	300 ± 31.2a		2.00 ± 0.02a	0.19 ± 0.07a		4.30 ± 1.09a	9.48 ± 0.69a	
DR-S0	1.91 ± 2.07A	50.8 ± 3.77C	132 ± 28.4C	1.96 ± 0.22A	0.64 ± 0.02A	0.18 ± 0.03A	1.54 ± 0.18A	6.89 ± 0.65A	8.19 ± 0.76B
DR-S1	2.19 ± 1.81A	186 ± 30.0B	280 ± 18.1B	2.42 ± 0.59A	0.55 ± 0.07A	0.16 ± 0.05A	1.60 ± 0.22A	6.44 ± 1.16A	8.80 ± 0.39AB
DR-S2	1.08 ± 2.03A	299 ± 18.1A	402 ± 8.16A	2.32 ± 0.53A	0.56 ± 0.29A	0.16 ± 0.05A	1.44 ± 0.14A	6.39 ± 0.64A	9.39 ± 0.29A

^*^Mean ± SD, Different lowercase and uppercase letters indicate significant differences (*P* < 0.05) between treatments in the UR and DR systems, respectively, according to Tukey’s multiple range tests.

**Table 3:** Annual CH_4_ and N_2_O emissions, grain yield, NGWP and GHGI of UR and DR systems over the three rotations from November 2008 to November 2011.

Treatment	CH_4_	N_2_O	Yield	SOCSR	NGWP	GHGI
	(kg ha^−1^)	(kg N ha^−1^)	(t ha^−1^)	(t ha^−1^)	(t CO_2_ ha^−1^)	(kg CO_2_ kg^−1^grain)
UR-S0	104 ± 10.7c	2.26 ± 0.18a	13.3 ± 0.59a	0.08 ± 0.41c	4.32 ± 0.28a	0.34 ± 0.03a
UR-S1	208 ± 25.7b	2.08 ± 0.18a	13.7 ± 0.97a	0.94 ± 0.09b	4.60 ± 0.82a	0.36 ± 0.05a
UR-S2	303 ± 31.6a	2.19 ± 0.09a	13.8 ± 1.66a	1.77 ± 0.20a	4.84 ± 1.05a	0.38 ± 0.09a
DR-S0	185 ± 28.3C*	2.77 ± 0.26A*	16.6 ± 0.20A***	0.47 ± 0.30C	5.87 ± 0.85C*	0.36 ± 0.07C
DR-S1	469 ± 35.0B***	3.13 ± 0.55A*	16.9 ± 1.29A*	1.43 ± 0.40B	12.2 ± 1.44B**	0.73 ± 0.07B**
DR-S2	702 ± 27.5A***	3.04 ± 0.40A*	17.2 ± 1.04A*	2.42 ± 0.39A	16.4 ± 0.92A***	0.97 ± 0.13A**

^*^Mean  ±  SD, Different lowercase and uppercase letters indicate significant differences (P < 0.05) between treatments in the UR and DR systems, respectively, according to Tukey’s multiple range tests. Asterisks indicate significant differences between the UR and DR systems for the same treatments. *P < 0.05; **P < 0.01. ***P < 0.001.

## References

[b1] IPCC. Climate Change 2013: The Physical Science Basis in *Contribution of Working Group I to the Fifth Assessment Report of the Intergovernmental Panel on Climate Change* (eds Stocker T.F. *et al.*) 710–716 (Cambridge and New York, 2013).

[b2] Robertson G. P., Paul E. A. & Harwood R. R. Greenhouse gases in intensive agriculture: contributions of individual gases to the radiative forcing of the atmosphere. Science 289, 1922–1925 (2000). 10988070 10.1126/science.289.5486.1922

[b3] Mosier A. R., Halvorson A. D., Reule C. A. & Liu X. J. Net global warming potential and greenhouse gas intensity in irrigated cropping systems in northeastern Colorado. J Environ Qual 35, 1584–1598 (2006). 16825479 10.2134/jeq2005.0232

[b4] Robertson G. P & Grace P. R. Greenhouse gas fluxes in tropical and temperate agriculture: the need for a full-cost accounting of global warming potentials. In Tropical Agriculture in Transition—Opportunities for Mitigating Greenhouse Gas Emissions? Springer Netherlands, 51–63 (2004).

[b5] Smith, P. *et al.* (2007) Agriculture. In: Climate Change 2007: Mitigation. Contribution of Working Group III to the Fourth Assessment Report of the Intergovernmental Panel on Climate Change. Cambridge University Press, Cambridge, pp 497 540

[b6] Cai Z. C. *et al.* Measurements of CH_4_ and N_2_O emissions from rice paddies in Fengqiu, China. Soil Sci Plant Nutr 45, 1–13 (1999).

[b7] Wang J. Y. *et al.* Water regime–nitrogen fertilizer–straw incorporation interaction: field study on nitrous oxide emissions from a rice agroecosystem in Nanjing, China. Agric Ecosyst Enviro 141, 437–446 (2011). 10.1016/j.jes.2017.06.007 29478650

[b8] FAOSTAT. *Download data - rice cultivation* (2015) Available at: http://faostat3.fao.org/faostat-gateway/go/to/download/G1/GR/E (Accessed: 1st June 2015).

[b9] Yan X. Y., Akiyama H., Yagi K. & Akimoto H. Global estimations of the inventory and mitigation potential of methane emissions from rice cultivation conducted using the 2006 Intergovernmental Panel on Climate Change Guidelines. Global Biogeochem Cycles 23, GB003299 (2009).

[b10] Zou J. W. *et al.* Changes in fertilizer-induced direct N_2_O emissions from paddy fields during rice-growing season in China between 1950s and 1990s. Glob Chang Biol 15, 229–242 (2009).

[b11] Frolking S. *et al.* Combining remote sensing and ground census data to develop new maps of the distribution of rice agriculture in China. Global Biogeochem Cycles 16, GB001425 (2002).

[b12] Zou J. W. *et al.* A 3-year field measurement of methane and nitrous oxide emissions from rice paddies in China: Effects of water regime, crop residue, and fertilizer application. Global Biogeochem Cycles 19, GB002401 (2005).

[b13] Zhang H. L. *et al.* Emissions of CH_4_ and N_2_O under different tillage systems from double-cropped paddy fields in Southern China. Plos One 8, e65277 (2013). 23750250 10.1371/journal.pone.0065277 PMC3672096

[b14] Shang Q.Y. *et al.* Net annual global warming potential and greenhouse gas intensity in Chinese double rice-cropping systems: a 3-year field measurement in long-term fertilizer experiments. Glob Chang Biol 17, 2196–2210 (2011).

[b15] Xiong Z. Q. *et al.* Measurement of nitrous oxide emissions from two rice-based cropping systems in China. Nutr Cycl Agroecosyst 64, 125–133 (2002).

[b16] Xing G. X. *et al.* Nitrous oxide emissions from paddy soil in three rice-based cropping systems in China. Nutr Cycl Agroecosyst 64, 135–143 (2002).

[b17] Zeng X. Y., Ma Y. T. & Ma L. R. Utilization of straw in biomass energy in China. Renew Sustain Ener 11, 976–987 (2007).

[b18] Gao L. W. *et al.* Estimation of nutrient resource quantity of crop straw and its utilization situation in China. Trans Chin Soc Agric Eng (Chin) 25, 173–179 (2009).

[b19] Shen J. L. *et al.* Contrasting effects of straw and straw-derived biochar amendments on greenhouse gas emissions within double rice cropping systems. Agric Ecosyst Enviro 188, 264–274 (2014).

[b20] Al-Kaisi M. M. & Yin X. H. Tillage and crop residue effects on soil carbon and carbon dioxide emission in corn–soybean rotations. J Environ Qual 34, 437–445 (2005). 15758095 10.2134/jeq2005.0437

[b21] Yao Z.S. *et al.* Tillage and crop residue management significantly affects N-trace gas emissions during the non-rice season of a subtropical rice-wheat rotation. Soil Biol Biochem 41, 2131–2140 (2009).

[b22] Ma J., Ma E. D., Xu H., Yagi K. & Cai Z.C. Wheat straw management affects CH_4_ and N_2_O emissions from rice fields. Soil Biol Biochem 41, 1022–1028 (2009).

[b23] Wang J. Y. *et al.* Methane emissions from a rice agroecosystem in South China: effects of water regime, straw incorporation and nitrogen fertilizer. Nutr Cycl Agroecosyst 93, 103–112 (2012).

[b24] Yao Z. S. *et al.* Nitrous oxide and methane fluxes from a rice-wheat crop rotation under wheat residue incorporation and no-tillage practices. Atmos Environ 79, 641–649 (2013).

[b25] Bayer C. *et al.* A seven-year study on the effects of fall soil tillage on yield-scaled greenhouse gas emission from flood irrigated rice in a humid subtropical climate. Soil Till Res 145, 118–125 (2015).

[b26] Yan X. Y., Cai Z.C., Ohara T. & Akimoto H. Methane emission from rice fields in mainland China: Amount and seasonal and spatial distribution. J Geophys Res : Atmos (1984–2012) 108, (D16) (2003).

[b27] Dong H. B. *et al.* Effect of ammonium-based, non-sulfate fertilizers on CH_4_ emissions from a paddy field with a typical Chinese water management regime. Atmos Environ 45, 1095–1101 (2011).

[b28] Cai Z. C. A category for estimate of CH_4_ emission from rice paddy fields in China. Nutr Cycl Agroecosyts 49, (1–3), 171–179 (1997).

[b29] Dobbie K. E & Smith K. A. Nitrous oxide emission factors for agricultural soils in Great Britain: The impact of soil water-filled pore space and other controlling variables. Glob Chang Biol 9, 204–218 (2003).

[b30] Ma Y. C. *et al.* Net global warming potential and greenhouse gas intensity of annual rice–wheat rotations with integrated soil-crop system management. Agric Ecosyst Enviro 164, 209–219 (2013).

[b31] Zhao X., Min J., Wang S. W., Shi W. M. & Xing G. X. Further understanding of nitrous oxide emission from paddy fields under rice/wheat rotation in south China. J Geophys Res: Biogeo (2005–2012) 116, (G2) (2011).

[b32] Schütz H., Seiler W. & Conrad R. Processes involved in formation and emission of methane in rice paddies. Biogeochemistry 7, 33–53 (1989).

[b33] Yan X.Y., Yagi K., Akiyama H. & Akimoto H. Statistical analysis of the major variables controlling methane emission from rice fields. Glob Chang Biol 11, 1131–1141 (2005).

[b34] Yao Z. S. *et al.* Effects of organic matter incorporation on nitrous oxide emissions from rice-wheat rotation ecosystems in China. Plant Soil 327, 315–330 (2010).

[b35] Khalil M. I., Rosenani A. B., Van Cleemput O., Fauziah C. I. & Shamshuddin J. Nitrous oxide emissions from an ultisol of the humid tropics under maize-groundnut rotation. J Environ Qual 31, 1071–1078 (2002). 12175023 10.2134/jeq2002.1071

[b36] Jiao Z. H. *et al.* Water management influencing methane and nitrous oxide emissions from rice field in relation to soil redox and microbial community. Commun Soil Sci Plant Anal 37, 1889–1903 (2006).

