# Supplementary material for: Differences in net global warming potential and greenhouse gas intensity between major rice-based cropping systems in China
Source: Sci Rep. 2015 Dec 2;5:17774. doi: 10.1038/srep17774 (PMC4667221; doi:10.1038/srep17774)
Supplement: Supplementary Materials [file srep17774-s1.pdf]

Supplementary material

Differences in net global warming potential and greenhouse gas intensity between major rice-based cropping systems in China

Running head: NGWP and GHGI of major rice systems

Zhengqin Xiong[1,]*, Yinglie Liu[1], Zhen Wu, Xiaolin Zhang, Pingli Liu, Taiqing Huang

Figure legends

Fig. S1. Daily mean air temperature (ºC) and precipitation (mm) during the three annual cycles from November 2008 to November 2011 in the experimental field.

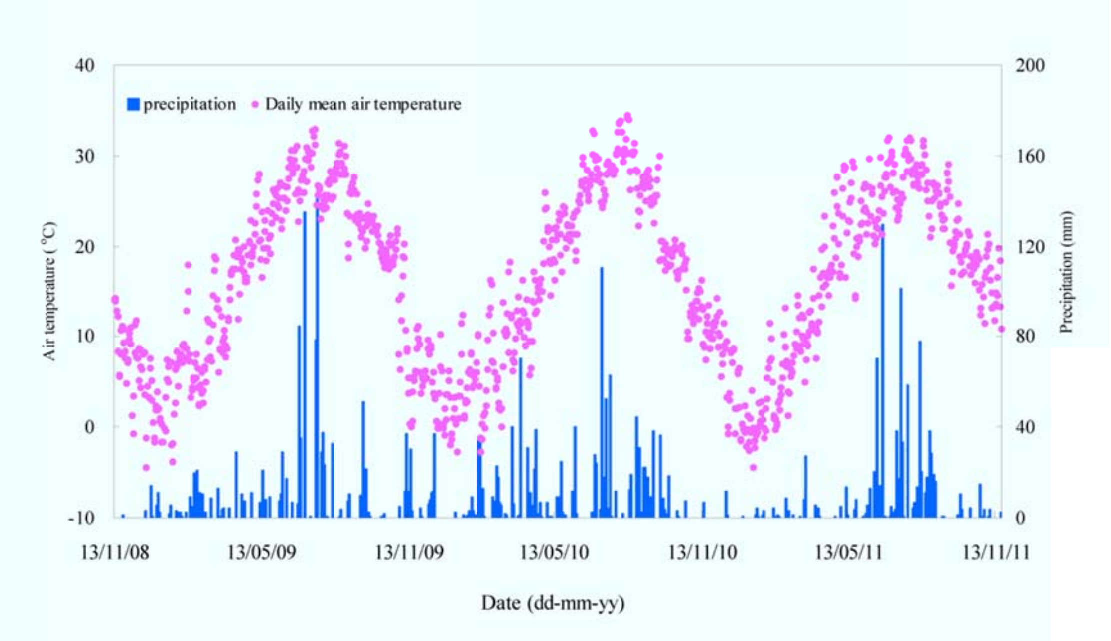

Figure S1

Table S1 Primary management practices for UR and DR systems from November 2008 to November 2011

		UR system		DR system		
Year	Management	Wheat	Rice	Oil rape	Early rice	Late rice
08–09	Straw incorporation		09/6/21		09/5/15	09/8/4
	Basal fertilizer	08/11/11	09/6/22	08/11/11	09/5/18	09/8/6
	Sowing/transplanting	08/11/11	09/6/22	08/11/11	09/5/18	09/8/6
	First topdressing	09/2/4	09/7/13	09/2/4	09/6/4	09/8/18
	Midseason drainage		09/7/29		09/6/13	09/9/5
	Second topdressing	09/3/7	09/8/7	09/3/7	09/6/23	09/9/12
	Harvest	09/5/27	09/10/24	09/5/15	09/8/4	09/11/5
09–10	Straw incorporation		10/6/18		10/5/19	10/8/8
	Basal fertilizer	09/11/13	10/6/20	09/11/13	10/5/26	10/8/12
	Sowing/transplanting	09/11/15	10/6/20	09/11/13	10/5/26	10/8/12
	First topdressing	10/2/21	10/7/6	10/2/21	10/6/8	10/8/29
	Midseason drainage		10/7/29		10/6/20	10/9/15
	Second topdressing	10/3/18	10/8/11	10/3/18	10/6/30	10/9/25
	Harvest	10/6/6	10/10/10	10/5/19	10/8/8	10/11/20
10–11	Straw incorporation		11/6/21		11/5/18	11/7/31
	Basal fertilizer	10/11/5	11/6/22	10/11/27	11/5/21	11/8/6
	Sowing/transplanting	10/11/6	11/6/22	10/11/27	11/5/22	11/8/6
	First topdressing	11/2/12	11/7/9	11/2/12	11/6/5	11/8/23
	Midseason drainage		11/7/27		11/6/26	11/9/6
	Second topdressing	11/3/18	11/8/6	11/3/18	11/7/6	11/9/18
	Harvest	11/6/1	11/10/30	11/5/18	11/7/31	11/11/20

Table S2 Nitrogen fertilizer and straw incorporation management for different treatments of UR and DR systems

Treatment	crop	N level (kg N ha^{-1})	Straw (t ha^{-1})	crop	N level (kg N ha^{-1})	Straw (t ha^{-1})	crop	N level (kg N ha^{-1})	Straw (t ha^{-1})
UR-S0	wheat	250	–	rice	250	0			
UR-S1	wheat	250	–	rice	250	3			
UR-S2	wheat	250	–	rice	250	6			
DR-S0	oil rape	250	–	early rice	200	0	late rice	200	0
DR-S1	oil rape	250	–	early rice	200	3	late rice	200	3
DR-S2	oil rape	250	–	early rice	200	6	late rice	200	6
